# Effects of football versus aerobic exercise training on muscle architecture in healthy men adults: a study protocol of a two-armed randomized controlled trial

**DOI:** 10.1186/s13063-020-04797-y

**Published:** 2020-12-09

**Authors:** Guevar Alkhateeb, Lars Donath

**Affiliations:** Department of Intervention Research in Exercise Training, German Sport University Cologne, 50933 Köln, Germany

**Keywords:** Muscle architecture, Patellar tendon, Football training, Aerobic training, Isokinetic dynamometer, Passive resisting torque, Randomized controlled trial

## Abstract

**Background:**

Sports and exercise training can attenuate age-related declines in physical function. As people age, they suffer a progressive deterioration of overall muscle structure and function, such as muscle diameter, strength, mass, and power. Therefore, supporting older adults—aged 50 years and above—to continue being physically active is a very important factor. Several forms of exercise (strength, agility, endurance, balance, and flexibility) are recommended. In this regard, football has been repeatedly shown to be an integrative approach to promote measures of strength, endurance, and agility. However, there has been no previous randomized controlled trial that comparatively investigates the effects of football training versus traditional aerobic exercise training on muscle architecture and patella tendon properties in healthy community dwellers. The study protocol is designed to examine whether football differentially affects muscle thickness, muscle length, fascicle length, pennation angle, patella tendon length, and thickness compared to a workload matched traditional aerobic exercise training regimen.

**Methods:**

The study sample consists of 60 untrained but healthy men (50–60 years old), who will be randomly assigned (strata: age, activate) to two groups: football group (*n* = 30) and aerobic group (*n* = 30). The intervention will take place within 12 consecutive weeks, two times a week for 60 min each session. The football group will perform recreational football training as a large-sided game, whereas the aerobic group undergoes a running exercise. Both groups have the same external workload ranging between moderate and high exercise intensity. The outcome measure will be collected before and after the intervention period.

**Discussion:**

Findings of this study will provide insight into the effects of 24 sessions of both football and aerobic training program on the selected groups of men adults, including detecting their effects on the thigh muscle architecture.

**Trial registration:**

DRKS—German Clinical Trials Register, DRKS00020536. Registered on 30 January 2020.

## Administrative information

Note: the numbers in curly brackets in this protocol refer to SPIRIT checklist item numbers. The order of the items has been modified to group similar items (see http://www.equator-network.org/reporting-guidelines/spirit-2013-statement-defining-standard-protocol-items-for-clinical-trials/).

**Table:** 

Title {1}	Effects of football versus aerobic exercise training on muscle architecture in healthy men adults: a study protocol of a two-armed randomized controlled trial
Trial registration {2a and 2b}.	German Clinical Trials Register (DRKS). Number: DRKS00020536
Protocol version {3}	Issue Date: 30 Jan 2020 Protocol Amendment Number: 05 Recruitment date: From 1/2/2020 until 15/03/2020 Author(s): Guevara Alkhateeb/ Lars Donath
Funding {4}	This research is partially funded by Tishreen University, and the first author. The publishing rights are owned by the authors.
Author details {5a}	Guevar Alkhateeb ^1^, Lars Donath ^2^ ^1^ Department of Intervention Research in Exercise Training, German Sport University Cologne, 50,933 Köln, Germany; Guevar.Alkhateeb@gmail.com ^2^ Department of Intervention Research in Exercise Training, German Sport University Cologne, 50,933 Köln, Germany; l.donath@dshs-koeln.de
Name and contact information for the trial sponsor {5b}	German Sport University Cologne – Institute of Training Science and Sport Informatics. Department of Intervention Research in Exercise Training Prof. Dr. Lars Donath, FECSS Am Sportpark Müngersdorf 6 50,933 Köln Germany
Role of sponsor {5c}	This is independent research and it does not have any sponsor.

## Introduction

### Background and rationale {6a}

The section is going to introduce a number of points in order: (a) the progressive deterioration aging has on muscles, (b) defining muscle architecture and identifying how training impacts it, (c) highlighting the benefits of football training and aerobic training on aging, and (d) the purpose of the study—to conduct a comparative assessment between the effects football training and aerobic training have on aging, and which one is better.

Besides impaired endurance and balance performance, declines in muscle strength greatly affect the progressive declines in physiological function with age. Good physiological function is maintained until middle age and thereafter progressively deteriorates [26] in the way that the overall muscular strength increased until the age of 30, and remains constant without change until about the age of 50, and then decreased by about 30% between the ages of 50–70 [38]. Also, muscle mass declines by 20 to 50% in humans between the ages of 40 and 80, which is accompanied by a decrease in muscle strength [6, 18]. Moreover, significant reduction of type II fast-twitch fibers is considered highly responsible for the decline in muscle cross-sectional area (CSA) [23]. However, annual strength decline from 50 years of age onwards amounts to 1.4 to 2.5% per year with an increasingly progressive loss of muscle fibers in the limbs [36, 39]. Almost half of the muscle fibers are reported to be lost by the age of 80 [18]. Therefore, middle-aged adults are required to have more physical activity in order to compensate for this continued decline. Adults play sport for a range of health-related and social reasons that can contribute to the experience of successful aging.

In this regard, muscle architecture is considered as an important contributor to the muscles’ overall function. Crucial parameters of muscle architecture include muscle mass, muscle fiber length, pennation angle (PA), and sarcomere length [53]. Also, there are other parameters that can be used in muscle architectural analysis like muscle length (ML), fiber length, and physiological CSA in addition to PA [34]. They play an important role in force generation. Muscle size and force production are strongly influenced by fascicle arrangement (i.e., muscle architecture) within the muscle [4]. Muscle architecture development is associated with running, squat movement, and jumping [25, 27]. Muscle thickness (MT) and PA along with shorter fascicles were beneficial for jumping ability at increased pre-stretch loads [2]. Moreover, training programs like strength training cause several changes to muscle architectures including MT, and these changes were related to relative strength and speed improvements [48].

Sports training plays an important role in the reduction of the age-related progressive deterioration of overall muscle structure and function. For example, 6 weeks of resistance training for older adults is effective at increasing strength, muscle quality, and muscle morphology [52]. Ensuring access to activities that can benefit social health is of great importance to older adults. As sport can provide participation opportunities across generations, it can be an ideal physical activity option for this age group [14]. Moreover, increased physical activity in middle age is probably followed by a reduction in mortality to the same level as seen among men with constantly high physical activity.

Recreational football can be an alternative exercise modality for untrained, healthy, or unhealthy middle-aged and older adults [22]. Football is an effective stimulant of musculoskeletal, metabolic, and cardiovascular adaptations of importance for health and thereby reduces the risk of developing lifestyle diseases [22]. Also, it has effects on various age categories and populations on muscular fitness, health profile, and physical capacity [29]. In this context, aerobic activity is efficient to improve physical fitness and functionality in adults. In fact, maintaining a healthy life would be more appropriate in step-aerobics exercises due to having higher tempos and rhythms than the run-walk exercises [35]. Moreover, aerobic program training can enhance muscle protein, synthesis, irrespective of age [21].

Several studies have recently focused on the effect of resistance or strength training programs on muscle architecture. There are no previous studies that have analyzed the effects of football training program or aerobic training program on muscle architecture, nor there is comparative study between football and aerobic training methods in terms of their effectiveness on muscle architecture. The study protocol is designed to investigate whether football differentially affects muscle architecture, work, power, torque, and patella tendon (PT) compared to traditional aerobic exercise training program in adult men.

### Objectives {7}

The main objective of this randomized controlled trial is to determine the effects of football versus traditional aerobic exercise training on muscle architecture, such as MT, ML, fascicle length (FL), PA, PT length, thigh muscle power, work, and torque. A secondary objective is to examine the effects of both training programs on aerobic capacity and explosive power.

### Trial design {8}

This study is a randomized controlled trial with parallel arms conducted and reported in accordance with CONSORT guidelines for non-pharmacologic treatment.

## Methods: participants, interventions, and outcomes

### Study setting {9}

The measurements will take place at the Departments of Radiology and Physical Therapy at Tishreen University Hospital. The intervention training programs will start from 15 April 2020 until 15 July 2020 in the Latakia’s Sports City Stadium. Sixty untrained men between 50 and 60 years old will be randomly assigned to experimental training groups, either a training group (*n* = 30) following football training program (FG) or a group (*n* = 30) following traditional aerobic training (AG). All participants need to be healthy, without presenting any cardiological, orthopedic, and neurological impairment that can affect testing and training (see inclusion and exclusion criteria for details).

Participants will be asked to complete health and neuromuscular problems or a disease history questionnaire and to provide written approval from their health care provider. The data can arise from two samples of participants where each participant is measured twice before and after an intervention. The measurements used in the protocol will be taken before and after the intervention period (12 weeks). The post-intervention test will be scheduled in the following week, after finishing the 12-week intervention period.

### Eligibility criteria {10}

Inclusion criteria are as follows: (1) healthy adult male volunteers between the ages of 50 and 60 years old, (2) participants must be in good health as determined by screening medical history and physical examination, and (3) they must not suffer from any neuromuscular, cardiovascular, or psychiatric disorder. The International Physical Activity Questionnaire (IPAQ) will be used to measure health-related physical activity in populations. IPAQ has reasonable measurement properties for monitoring population levels of physical activity among 18–65 years old adults in diverse settings [15]. The questions are about the time you spent being physically active in the last 7 days. They include questions about activities you do at work, as part of your house and yard work, to get from place to place, and in your spare time for. The long, self-administered IPAQ questionnaire has acceptable validity [42].

Exclusion criteria are as follows: (1) participants who have a significant history of alcoholism or drug/chemical abuse within the last 2 years; (2) participants with positive results on tests for drugs of abuse or alcohol at screening and check-in; (3) history of unstable psychiatric disorder requiring medications or hospitalization within the previous 12 months; (4) history of a concurrent illness that required hospitalization within 14 days prior to day 1 of the study; (5) any condition that in the clinical judgment of the investigator would make the subject unsuitable for participation; (6) participants who have had a clinically significant illness within 4 weeks prior to day 1; (7) history or current evidence of clinically significant hepatic, renal, cardiovascular (i.e., deep venous thrombosis, pulmonary embolism), psychological, pulmonary, metabolic, endocrine, neurologic (i.e., transient ischemic attack or stroke within the past 6 months), infectious, gastrointestinal (i.e., any condition which may affect drug absorption), hematologic, and oncologic disease, retinopathy, or other medical disorders; (8) history of unexplained syncope; and (9) participants who, in the opinion of the investigator, should not participate in the study.

### Who will take informed consent? {26a}

All participants will be informed of all research protocols and details of the training program, including the applied load intensity. The first author (G.A.) will discuss the trial with participants in light of the information provided in the information sheet. We will obtain written consent from patients willing to participate in the trial.

### Additional consent provisions for collection and use of participant data and biological specimens {26b}

On the consent form, participants will be asked if they agree to the use of their data should they choose to withdraw from the trial. Participants will also be asked for permission for the research team to share relevant data with people from the universities taking part in the research or from regulatory authorities, where relevant. The data will be possible for sharing with people from the universities after the permission of participants.

## Interventions

### Explanation for the choice of comparators {6b}

In spite of the increasing numbers of training program research for seniors, many age-related diseases are considered as serious factors that could threaten their health and life. Our research aims to discover the effects of two different training program methods in order to delay the appearance of retraction of muscle properties.

### Intervention description {11a}

The training program will be carried out in two groups at the sports city training stadium (Latakia, Syria), two times a week (60 min per session) on non-consecutive days for more than 12 weeks (24 sessions). Both groups will have the same session load. According to Monoem et al. [37], football players under 18 years old practice small- or large-sided games with the average heart rate that is 155 to 167 beats per minute (bpm) and maximum heart rate (HRMAX) that is between 70 and 90%. To determine training load, the RPE will be obtained using the 10-point Borg scale by having participants rating their training perceived effort 30 min after the end of training according to the procedures suggested by Foster [19].

During the familiarization session, each subject will be given instructions on the use of the RPE scale (Table 1). We will ask the participants to use any number on the scale to rate their overall effort. A rating of 0 indicates no effort (rest), and a rating of 10 indicates maximal effort and associates with the most stressful exercise ever performed. Following each set during the training, the subject will be asked “How would you rate your effort?” We will use the session RPE measure to rate the entire workout (Table 1). A single arbitrary unit representing the magnitude of global TL for each session is then calculated by the multiplication of training intensity and the length of training (min). TL(A.U.) = RPE × session duration (min), where TL is the training load, A.U. is the arbitrary units, and RPE is the rating of RPE.

**Table 1:** RPE scale

**Descriptor**	Very easy		Easy	Moderate	Somewhat hard			Hard		Very hard	Maximal
**Rating**	1	2	3	4	5	6	7	8	9	10	

All sessions will include 5 min of warm-up at the beginning of the training session and 5 min cooldown at the end of it, for both groups. The warm-up will focus on pulse-raising, mobility, and preparatory stretching for the conditioning component with joint mobility (starting at the top of the body and working the way down, from the neck, shoulders, upper back, hips, and ankles) and continuous leg movement to facilitate the venous return and coordination exercises (toe-tapping arm circles, heel walks, high knee walk, backward high knee skip). Cooldown will include transition from conditioning component to the stretching phase, which comprises static and dynamic stretching exercises for all major muscle groups. Sessions will be deemed completed when at least 90% of the prescribed exercises have been successfully performed.

Considering that FIFA11+ exercises aim to prevent injury, and that the target group age is between 50 and 60 years old, participants in both groups will perform FIFA 11+ and functional training (4–6) exercises using the major muscle groups with low and moderate intensity in a way that the load did not affect our program goals. Running exercises at low/moderate speed will be combined with planting/cutting movements (5 min). The exercises will be as follows: running exercises (straight ahead, hip out, hip in, circling partner, shoulder contact, quick forwards and backwards) and strength, balance, and agility (sideways bench with leg, vertical hump, jumping box and lateral jumps, across the pitch, running plant and cut bounding, running with toe raise, squats one leg squad, squats walking lunges, hold the ball, single leg test your partner, single-leg throwing the ball with partner, hamstring). Every single exercise has 2–4 sets with 4–8 repletion, with 40 s rest break. At the same time, participants in FG will perform the same training characteristics.

In the main part of the training, we will focus on small game running (20–30–40 m) × 5–10 with 3–5 sets and 1–2 min rest between sets for (AG). Also in the same training load, FG will perform competitive activities like small- or large-sided game or a football match (4 vs 4, 5 vs 5, etc.). All participants in both groups will work in the same/similar conditions and rules. None of the participants will play as a goalkeeper in the FG (Table 2). The level of effort for muscle-strengthening activities should be between moderate and high. On a 10-point scale, where no movement is 0, and maximal effort of a muscle group is 10, the moderate-intensity effort is a 5 or 6 and high-intensity effort is a 7 or 8 (the ACSM guideline recommendations have been considered) Table 2.

**Table 2:** Exemplary training programs for the aerobic and football group

Training time	FG	AG	Intensity
5 min	Warm-up	Warm-up	Low
15 min	Injury prevention (FIFA11 ± functional training)	Injury prevention (FIFA11 ± functional training)	Moderate
35 min	Football match (competition activities)	Aerobic training (running)	Moderate to high
5 min	Cooldown	Cooldown	Low

Football players practice small-sided games (3 vs 3, 4 vs 4, 5 vs 5, 6 vs 6) with the average heart rate of 155 to 167 bpm [12]. The intensity of small- or large-sided game is moderate and high (the percentage of HRMAX is between 70 and 85%). Both groups will have the same session load (Table 3).

**Table 3:** Training session volume and load

	Time	Sets	Rep	Rest	Effort point scale
Warm-up	5 min				3–4
Injury prevention	15 min	2–4	4–8	40 s	4–5
Main part	35 min	3–5	5–10	1–2 min	7–10
Cooling	5 min	–	–	–	1–2

### Criteria for discontinuing or modifying allocated interventions {11b}

Any participant will be excluded as soon as he gets a serious physically injury or if he is unable to follow up on the training sessions for any other reasons.

### Strategies to improve adherence to interventions {11c}

The adherence reminder sessions will take place 15 min before every training session. This session will include the following:

- The importance of following a training program for general health.
- Instructions about how to practice carefully and how to avoid physical collision between participants, especially in competitions or games.
- The importance of adherence in order to develop the performance of the body, and the negative effects of absence from any training session.
- Instructions about the purpose of the study and the training plan.
- Instructions the importance of informing the coach about any injury or physical pain.
- Participants will have an opportunity to ask questions throughout the training program at any time.

Before the starting of every single month of the training program, there will be a special recreational trip in order to increase the motivation and commitment of participants.

### Relevant concomitant care permitted or prohibited during the trial {11d}

- Participants are allowed to take an additional break in the first training sessions.
- Participants should not eat a meal at least 2 h before the exercise.
- Participants should not use any additional assistive equipment during exercise.

### Provisions for post-trial care {30}

Although health insurance is free of charge in Syria, the first author will be responsible for any injury that occurs during the training sessions, and he will sign a paper for each participant proving his commitment to providing any medical treatment throughout and after the intervention period in coordination with Tishreen University Hospital.

### Outcomes {12}

#### Primary outcome: muscle architecture, tendon length, tendon thickness, muscle power, torque, and work

Muscle CSA, FL, MT, and PA will be examined with ultrasound imaging (Esaote, Technos MPX) in B-mode with a 10-MHz linear array transducer. This technique uses sound waves at fixed frequencies to create real-time images of the limb musculature in vivo (Esaote, Technos MPX, ultrasound). Conventional B-mode ultrasound can provide both quantitative and qualitative information on skeletal muscle tendons and ligaments [50]. Muscle ultrasound is a reliable method for the measurement of human FL, PA [33], and MT in B-mode images [46]. Also, ultrasound is a reliable and valid tool for the assessment of muscle size in older adults, ICC scores ranging from 0.92 to 0.999 [11].

Each of muscle power, torque, and work will be measured using isokinetic dynamometer test. An isokinetic dynamometer (Genu3 Easytech, Florence, Italy) will be used to estimate knee range of motion (ROM), passive resistive torque, power, and work for thigh muscles in an angular speed of 90° per second. Test-retest reliability will take place 2 weeks before the intervention (the same test twice over a period of time to a group of individuals). The scores from time 1 and time 2 can then be correlated in order to evaluate the test for stability over time. The Pearson product-moment correlation coefficient will be used to measure the strength of the linear relationship between the variables. Three to four submaximal practice trials will be given before each of the assessments. Knee extensor and flexor muscle power and work will be measured as the peak torque obtained over 3 trials during an isokinetic contraction using the passive concentric mode to test throughout the entire range of motion. Passive resistive torque will be obtained over 3 repetitions at the same speed degree. Peak torque will be gravity corrected using the Genu3 Easytech software.

#### Secondary outcomes: explosive power, aerobic capacity, and tendon CSA and length

The explosive power will be measured by using vertical jump test. The test is often used as a measurement of lower-body power [44] and thus as an indirect measure of performance. Vertical jump testing has been shown to be a valid and reliable measure of lower-body explosive power [44].

The high-intensity aerobic capacity will be measured by the YYIR tests. The test is a simple method for examining an athlete’s capacity to perform a repetitive high-intensity aerobic exercise [9]. Yo-Yo test has been repeatedly proven as reliable (ICC = 0.86 to 0.95) [49], and also, YYIR1 has been proven as a valid and reliable tool with high reproducibility for measuring high-intensity aerobic capacity from various sports and competition levels [16, 27], and a moderately reliable predictor of VO2 max [44, 49].

Tendon CSA and length will be measured using ultrasound imaging (Esaote, Technos MPX) in B-mode with a 10-MHz linear array transducer the same as muscle architecture measurements.

The muscle architecture and PT length measurements will take place from 1 March 2020 until 10 March 2020 for both groups in Tishreen University Hospital (Radiology Department). After that, the measurements of torque, power, and work will take place until 16 March 2020 in Tishreen University Hospital (Physical Therapy Department). Explosive power and aerobic capacity measurements will start directly after that until 20 March 2020 at Latakia Sports City Stadium.

### Participant timeline {13}

**Fig. 1:**
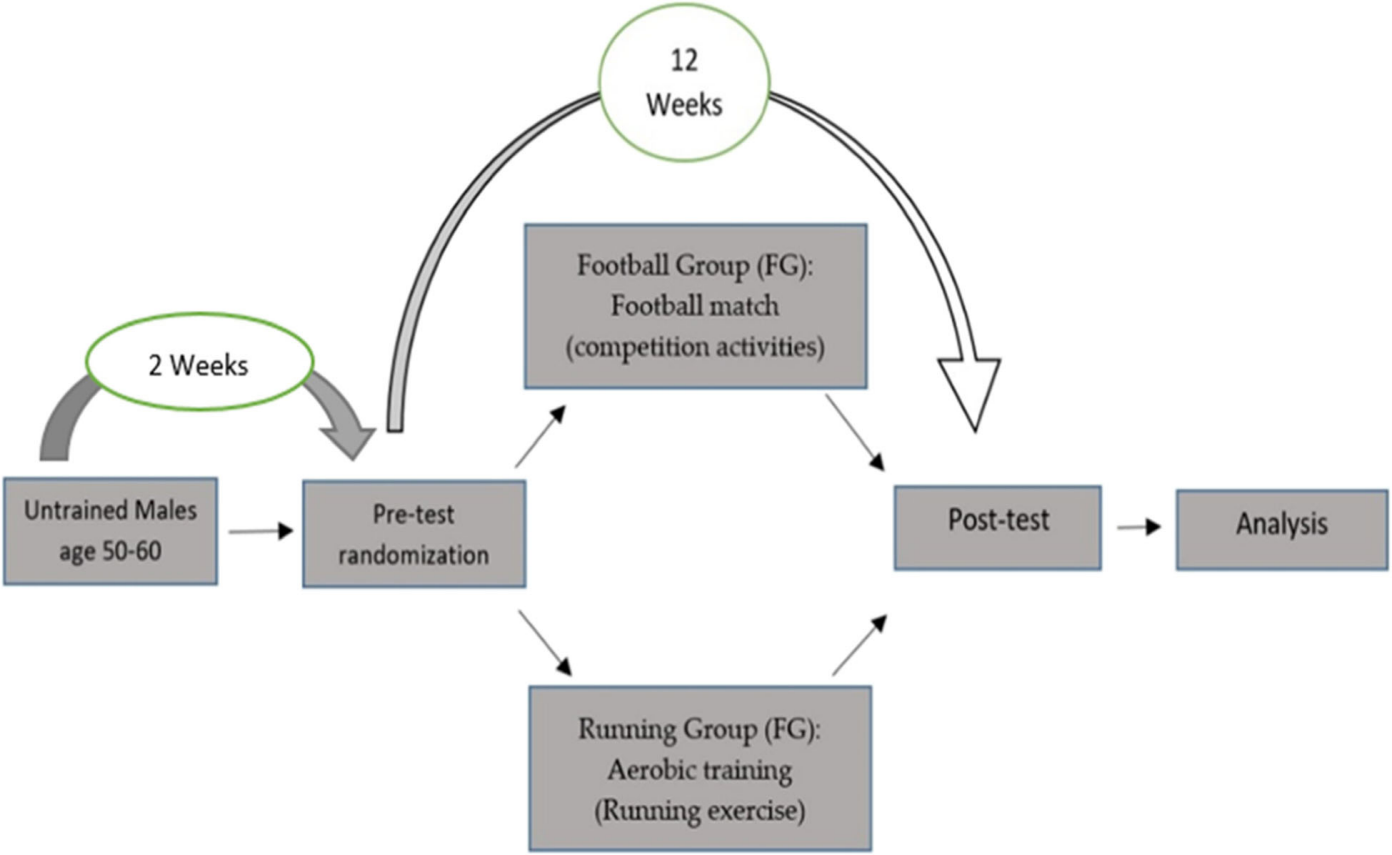
Participant timeline and study design

Participants will be assessed according to their medical reports from 1 February 2020. After meeting all the eligibility for recruitment, we will make appointments for the pre-tests in the next 20 days. The training program will take place from 15 April 2020 (Fig. 1).

### The schedule of enrolment, interventions, and assessments

Table 4 shows the schedule.

**Table 4:** The schedule of enrolment, interventions, and assessments

Post-allocation study period (weeks)															
	Enrolment	Baseline	Post-allocation												Follow-up
Timepoint**	***2 weeks***	0	***1***	***2***	***3***	***4***	***5***	***6***	***7***	***8***	***9***	***10***	***11***	***12***	
**Enrolment**															
**Eligibility screen**	X														
**Informed consent**	X														
**Allocation**		X													
**Interventions**															
***Football group***			X	X	X	X	X	X	X	X	X	X	X	X	
***Aerobic group***			X	X	X	X	X	X	X	X	X	X	X	X	
**Assessments**															
***VO2 max, explosive power***		X													X
***Range of motion, muscular power, and work***		X													X
***Muscle architecture (CSA, FL, MT, PA) and PT length***		X													X

### Sample size {14}

The sample size was calculated by using “Gpower” software 3.1.9.2. For the determination of the effect size, we made a pilot study which included 10 participants from every single group. We measured the PA of VM for both groups. The mean (M) for football group was 10.59 and 8.17 for AG. Also, the standard deviation (SD) for FG was 2.80 and 2.01 for aerobic one. Based on this, the effect size was 0.992. The α level was 0.05, and the power was 0.95. The minimum numbers of sample were 46 participants.

We raise the sample to 60 for better result. Sixty untrained but healthy adult men are equally divided into two groups (mean ± SD age 55 ± 5). This sample includes a dropout of 15% and provides a moderately detectable change between a group with an alpha level of *p* < 0.05. Some of them had experienced some of the age-related neuromuscular problems or diseases, at least within the last year.

### Recruitment {15}

Volunteers will be recruited through posters published in the official newspapers in the Latakia City, as well as via social media. Recruitment will finish once the number of volunteers required is complete. There will be no paid incentives to participate in this study, but the researcher will be responsible for all transportation costs and things necessary for sports (shoes, uniforms, water bottles).

## Assignment of interventions: allocation

### Sequence generation {16a}

The random allocation sequence will be created in permuted blocks of four and six using random allocation software version 1.0.0 [47].

### Concealment mechanism {16b}

Allocation concealment will be achieved using sequentially numbered, opaque sealed envelopes [17]. Participants will be assigned in two intervention groups: a football group and aerobic group in a 1:1 ratio. Allocation concealment will be ensured. The randomization code will not release until the participants have been recruited into the trial, which takes place after all baseline measurements have been completed.

### Implementation {16c}

The responsible committee for recruitment will send a form to the independent researcher who is not involved in any assessment of the outcome of the study. This form will include a randomization number with closed envelopes. The responsible staff for recruitment is not allowed to receive information about group allocation.

All participants who give consent for participation and who fulfill the inclusion criteria will be randomized. Randomization will be requested by an employer who is responsible for this process from Tishreen University by using randomization software [17]. Each participant of the trial will be given a unique numeric code with the three code length character by the independent researcher.

## Assignment of interventions: blinding

### Who will be blinded {17a}

Due to the nature of the intervention, neither participants nor staff can be blinded to allocation, but are strongly encouraged not to disclose the allocation status of the participant at the follow-up assessments.

### Procedure for unblinding if needed {17b}

The design is open label with only outcome assessors being blinded, so unbinding will not occur.

## Data collection and management

### Plans for assessment and collection of outcomes {18a}

#### Ultrasonography

Participants will report to the Human Performance Laboratory for ultrasound measurements after 72 h without any vigorous physical activity. They will be instructed to lay supine for 15 min to allow fluid shifts to occur before image collection. A 10-MHz linear probe scanning head (LA523 13-4) will be used to optimize spatial resolution and coat with water-soluble transmission gel and positioned on the skin surface to provide acoustic contact without depressing the dermal layer to collect the image. The length of the muscle fascicles and the PA, which is the angle of insertion of the fascicles into the muscle’s aponeurosis, represent the main structural parameters that can be detected via the conventional B-mode ultrasound modality [41]. Measures of CSA and FL will be obtained using a sweep of the muscle in the extended field of view mode with the gain set to 50 dB and image depth to 5 cm. The FL of rectus femoris (RF) and VM will be measured using “the intercept method” with this formula: FL = visible FL + predicted length [13].

All measures will be in the RF, vastus lateralis (VL), VM, and PT of the dominant leg. Subsequent measures will be taken using the same limb positioning and anatomical site and will be performed by the same examiner. After scanning, all images will be analyzed with image analysis software (ImageJ, version 1.45s), which quantifies muscle quality in the form of echo intensity (EI). A known distance of 1 cm as shown in the image will be used to calibrate the software program [13]. For the measures of RF and VM, the participant will be placed supine on an examination table, according to the American Institute of Ultrasound in Medicine, with the legs extended but relaxed and with a rolled towel beneath the popliteal fossa allowing for a 10 bend in the knee as measured by a goniometer. For measures of the VL and VM, participants will be placed on their non-dominant leg side with the legs together and relax to allow a 10 bend in the knee as measured by a goniometer. The measurement of RF CSA will be taken in the sagittal plane parallel to the long axis of the femur, and scanning occurs in the axial plane perpendicular to the tissue interface at 50% of the thigh length. Fifty percent of thigh length is determined as halfway from the anteroinferior iliac spine to the proximal border of the patella. VL and VM CSA measure at 50% of the distance from the most prominent point of the greater trochanter to the insertion. For all muscles, consecutive CSA images will be analyzed using the polygon tracking tool in the ImageJ software to obtain as much lean muscle as possible without any surrounding bone or fascia.

#### FL

FL will be measured using the extended field-of-view mode. A longitudinal sweep begins near the distal insertion along the muscle and continues towards the proximal head of the muscle. Fascicle length is determined by identifying a clear fascicle that extends continuously from the superficial aponeurosis to the deep aponeurosis.

#### MT

Measures of MT will be the same as described for CSA but with the probe oriented longitudinally to the muscle tissue interface for the three muscles. Within each muscle, MT measures perpendicularly from the superficial aponeurosis to the deep aponeurosis.

#### PA

The PA of the rectus femoris, vastuss lateralis, and vastus medialis will be measured using B-mode ultrasound at the same site as MT and CSA [1]. The transducer will be longitudinal to the muscle tissue interface, and 1 image will be analyzed offline. Muscle fiber will be determined as the intersection of the fascicles with the deep aponeurosis.

PT length will be measured via ultrasound scans (Esaote, Technos MPX, ultrasound) at knee joint angle of 90° (0° corresponding to full extension), as the distance between tendon insertions on the patella apex and the tibial tuberosity. Tendon CSA will be measured from transversal ultrasound scans (linear array transducer 5 cm, LA523, 10 MHz transducer). The reliability of this technique has been shown previously [45]. PT CSA is obtained as the average of two measurements performed at the proximal (CSAp) and distal (CSAd) insertions and at tendon mid-length (CSAm). The mean CSA of these three scans’ positions will be used for further analysis. PT length will be measured as the distance between the proximal insertion of the PT and the tibial insertion. The use of ultrasound to perform quantitative measurements of musculoskeletal tissues has shown reliability (tendon CSA: ICC of 0.91–0.98 and 0.96–0.98 for tendon length) [20].

#### Range of motion measurement

To determine the range of motion (ROM), an isokinetic dynamometer (Genu3 Easytech, Florence, Italy) will be used. Participants will be seated with a hip joint angle of 110°, and their knee will fully extend on the dynamometer. Moreover, participants will be secured with a strap on the upper body to exclude any evasive movement. The foot will be fixed with a strap to the footplate of the dynamometer. Moreover, the upper leg will be fixed with a strap to avoid any movement of the knee. Participants will first move to the neutral knee joint position in the dynamometer (90°). They will then regulate the motor of the dynamometer to get into dorsiflexion (stretching) position until the point of discomfort is reached.

#### Passive resistive torque

Participants will be positioned with their hips at approximately 80° (to minimize hamstring stiffness) and their knees at a maximal extension. Stabilization straps are placed over the knee, foot, and ankle. The knee moves passively through six continuous cycles between 10° plantar flexion and 10° dorsiflexion at 90°/s, with neutral (0°) being the line of the tibia perpendicular to the footplate. The participants will be instructed to do the maximum plantar and dorsiflexion. Visual analysis of the curves will demonstrate consistent torque profiles, which indicate passive resistive torque.

#### Isokinetic muscular power and work test

The power of knee extensor and flexor muscles will be measured in a sitting position using isokinetic dynamometers (Genu3 Easytech, Florence, Italy). After warm-up exercises, patients perform 4 maximal concentric strokes at an angular speed of 90° per second (knee extension flexion; ROM, 90–0°). Isokinetic tests will be performed when patients have no pain or swelling with ROM more than 120° of flexion with full extension. To ensure maximal effort, 3 to 4 training sessions are allowed before performing the test.

#### Vertical jump test

The subject should, therefore, be instructed to jump as high as possible and land on the same spot. If the subject deviates too far from this point (a landing area/spot can be marked out), then the result should be disregarded and the jump repeated. Once the test configuration has been set up and the subject and assessor are ready, then the test will begin: the participants should walk into the take-off/landing area and, when instructed to by the assessor, jump as high as they can and land on the same spot. At the peak of their jump height, they should hit the vanes of the Vertec device to displace as many of them as possible. This should be repeated so that the athlete completes 3 jumps in total. This way, an average or best of the three jumps can be calculated. Jump height is determined by the difference between the participants’ standing reach height and the highest vane displaced. The results of the test are reported in terms of jump height (cm). To ensure a valid and reliable result, it is a good practice to take an average of the three jumps performed by the athlete. The following formula can be used to calculate the average jump height: Average jump height = (jump 1 + jump 2 + jump 3)/3. Alternatively, the best score (i.e., highest jump) out of the three jumps can also be used.

#### Yo-Yo intermittent recovery test

Once the configuration of the tests has been set up, test principal investigator/officials are positioned at both shuttle lines (cone B and C), and participants are ready, then the test will begin. Participants begin the test from cone B. When instructed by the audio player, they must run towards cone C (this must be reached before the following beep signal) and immediately return to cone B before the next signal. Once cone B is reached, participants then have a 10-s recovery period in which they must jog from cone B towards cone A, and then back to cone B before the commencement of the next shuttle. In this test, the participants are only allowed two consecutive failed attempts before they are withdrawn from the test. That being, if the individual fails to reach cone C and back to cone B in the allocated time, one fail is issued. If this happens a second consecutive time, then they are eliminated. Once withdrawn from the test, the individual’s score must be recorded. The YYIR1 typically lasts for 5–15 min [28]. The test is comprised of 91 shuttles and can last up to approximately 29 min; however, it is very unlikely somebody will complete it. Scores can be presented in three ways: (1) total distance (meters), (2) level achieved, or (3) VO2 max. Total distance is more simple to understand and calculate, whereas level achieved is more complex as the test begins at level 5 and then skips to level 9 at the beginning.

### Plans to promote participant retention and complete follow-up {18b}

After confirming the commitment of the participants and obtaining papers proving their consent to participate, the responsible researcher will send reminders to attend their sessions through text messages, WhatsApp messages, and phone calls. All the participants can contact the intervention coordinator and the responsible researcher 24 h a day by calling their cell phones.

### Data management {19}

Data will be stored in the Departments of Radiology and Physical Therapy at Tishreen University Hospital with a password-protected computer file, to which only researchers will have access. Throughout the duration of the research study, the file password will be changed once a month. The first author will have a backup copy of all the information.

### Confidentiality {27}

Files with participant’s information will be coded with individual identification codes.

### Plans for collection, laboratory evaluation, and storage of biological specimens for genetic or molecular analysis in this trial/future use {33}

The trial does not involve any genetic or molecular analysis of biological specimens derived from humans. Our muscle measurements will be done in the Radiology Department and Physical Therapy Department at Tishreen University Hospital, and the data will be stored in a protected file on secure computer in the Hospital. Files with participant’s information will be coded with individual identification codes.

## Statistical methods

### Statistical methods for primary and secondary outcomes {20a}

We will use the SPSS V20 for statistical data analysis. First of all, we will calculate “mean separation” as a comparison for every means. The objective is to identify where and how many differences exist. Normality tests (SPSS Explore) will be used to determine if the data is well modeled by a normal distribution, and to compute how likely it is for a random variable. Paired samples *t* test will be used to compare the mean of every group to measure the effects of every single training method. Independent samples *t* test will be used to compare the mean between the football and aerobic groups before and after the intervention.

### Interim analyses {21b}

This is not applicable.

### Methods for additional analyses (e.g., subgroup analyses) {20b}

The statistical analysis is performed by an independent statistician, blinded for the group allocation by the end of the retest procedure. Perhaps there will be none, the group is not large but perhaps you are looking at differences based on area men live, if they previously played team football, etc.

### Methods in analysis to handle protocol non-adherence and any statistical methods to handle missing data {20c}

#### Regression imputation [24]

Imputation is the process of replacing the missing data with estimated values. Instead of deleting any case that has any missing value, this approach preserves all cases by replacing the missing data with a probable value estimated by other available information. After all missing values have been replaced by this approach, the dataset is analyzed using the standard techniques for a complete data.

### Plans to give access to the full protocol, participant-level data, and statistical code {31c}

Data sharing statement is no later than 3 years after the collection of the 1-year post-randomization interviews. We will deliver a completely identified dataset to an appropriate data archive for sharing purposes. The datasets analyzed during the current study are available from the corresponding author on reasonable request.

## Oversight and monitoring

### Composition of the coordinating center and trial steering committee {5d}

#### Organizational structure and responsibilities

##### First author and the second one (the supervisor)

- Preparation of protocol and revisions.
- Application procedures for obtaining approval of the Research Ethics Committee.
- Implementation of modifications required for the research registration and obtain the required approval from the Ethics Committee of Scientific Research.
- Publication of study reports.
- The supervisor will be responsible for reviewing the progress of the study and if necessary agreeing changes to the protocol.

##### Trial steering committee

The committee will be formed by the Scientific Research Department of Tishreen University. Tishreen University research supervisor will be nominated as a coordinator. The steering committee should monitor trial progress and conduct and advise on scientific credibility. It will consider and act, as appropriate, upon the recommendations of the Ethics of Scientific Research Committee at Tishreen University and ultimately carries the responsibility for deciding whether a trial needs to be stopped on grounds of safety or efficacy. It will be observing the recruitment of participants with liaising with the principal researcher and reviewing the progress of the study and if necessary agreeing changes to the protocol brochure to facilitate the smooth running of the study. The steering committee will meet once every 2 weeks to discuss the reports and the monitoring process. There will be no Stakeholder and Public Involvement Group.

### Composition of the data monitoring committee, its role and reporting structure {21a}

There is no need for a formal data monitoring committee because of the short duration of the training program and the known minimal risks.

### Adverse event reporting and harms {22}

The adverse event will be defined as any serious injury that occurred in a subject without regard to the possibility of a causal relationship. Adverse events will be collected after the subject has provided consent and enrolled in the study.

If a subject experiences an adverse event after the informed consent document is signed (entry) but the subject has not started to receive study intervention, the event will be reported as not related to study drug. If training is discontinued as a result of an adverse event, study personnel will document the circumstances and data leading to discontinuation of the exercise.

Investigators will determine the relatedness of an event to study protocol based on the physical health situation for the subject before beginning the training program and the temporal relationship to the study training.

### Frequency and plans for auditing trial conduct {23}

Project Management Group will meet 4 days a week, before every single training. The Trial Steering Group and the Ethics Committee will meet two times per month to review conduct throughout the trial period.

### Plans for communicating important protocol amendments to relevant parties (e.g., trial participants, ethical committees) {25}

Any modification of the research procedures including changes of study objectives, study design, participant population, sample sizes, study procedures, or significant administrative aspects will lead to a change in the study protocol.

It will be under the supervision of the responsible professor and approved by the Ethics Committee of Tishreen University prior to implementation.

## Dissemination plans {31a}

This research is a part of a Ph.D. thesis, and the publishing rights are owned by the authors. The results will be presented several times to the supervisor professor and resident committee at German Sport University. Additional dissemination will occur through presentations at conferences, such as science education conferences, regionally and nationally, and through articles published in peer-reviewed journals.

The first author is delegated from Tishreen University to obtain a doctorate degree, and therefore, the university will be responsible only for providing the facilities and logistical support to complete the research.

The first author is a Ph.D candidate at the German Sport University Cologne, and all the results and researches related to the trial will only be published through it. The recruitment has been ended on 31 March.

## Discussion

Previous research has demonstrated that practicing small-sided recreational soccer games is an effective health-promoting activity for both untrained men and women. In untrained premenopausal women, thirty-seven participants were randomized to two training groups—football and running—training 1 h with equal average heart rates for 16 weeks (twice a week). The results show that there are effects on both left ventricular systolic and diastolic function and the training-induced cardiac adaptations appeared to be more consistent after football training compared with running [7]. In the same way, fifty healthy women were matched and randomized to a football (*n* = 25) or a running (RG, *n* = 25) group and compared with a control group with no physical training. After 16 weeks, resting heart rate was lowered, maximal oxygen uptake was elevated, and total fat mass decreased [32].

For untrained men, Krustrup et al. [30] have shown that recreational soccer training, organized as small-sided drills, has significant beneficial effects on health profile and physical capacity. In a study of some aspects, it is superior to frequent moderate-intensity running training, after training performed for 1 h (two or three times per week) for 12 weeks, at an average heart rate of 82% and 82% (1%) of HR (max) for 36 healthy men in football group and running group. Recreational football stimulates both aerobic and anaerobic energy turnover and is an effective type of training leading to significant cardiovascular and muscular adaptations as well as performance enhancements throughout a 12-week training period [31]. Moreover, positive adaptations in cardiovascular fitness obtained over 12 weeks of regular recreational football training with further development in musculoskeletal fitness [43]. Also, Andersen et al. [8] have investigated whether football has favorable effects in the treatment of mild-to-moderate arterial hypertension. The study included 25 untrained males aged 31–54 years. They were randomized to a football training group and a control group for 3 months. The results show that football as a treatment for hypertension can be an attractive non-pharmacological supplement to the treatment of mild-to-moderate arterial hypertension.

On the other hand, Timmins et al. [51] studied the architectural adaptation of the long head of the biceps femoris after concentric or eccentric strength training in the adaptation period of training and detraining. The results show that short-term strength training can contribute to the architectural change in the long head of the biceps femoris. Narici et al. [40] examined the impact of aging on muscle architecture of the medial gastrocnemius in 14 young and 16 elderly physically active people, who have had the same height, body weight, and physical activity regimen. Young people varied by age from 27 to 42 years and elderly people were 70–81 years old. Anatomical CSA and the volume of the medial gastrocnemius muscle were measured by computed tomography, and the length of the beams and PA were measured by ultrasound. Anatomical CSA and the amount of muscle in the elderly amounted to 19.1%, while in the young participants 25.4%. FL and PA also were smaller in the elderly group (10.2 and 13.2%, respectively). Another study investigated whether increased tendon-aponeurosis stiffness and contractile strength of the triceps surae muscle-tendon units induced by resistance training would affect running economy. This study was conducted on an exercise group (*n* = 13) which performed a 14-week exercise program. The results show after resistant training intervention an increase in maximum plantarflexion muscle strength (∼ 7%) and an increase in triceps surae tendon-aponeurosis stiffness (∼ 16%) [3].

Another study [10] investigated the effects of sports training on muscle architecture; 8 female and 15 male athletes performed 4 weeks of sprint, jump, and resistance training in addition to their sports training (standardization) before adopting one of three different sports programs. Muscle size, fascicle angle, and FL of the VL and RF muscles (using ultrasound procedures) as well as 20-m sprint run, vertical jump, and strength performance changes were examined. Significant muscle size and architectural adaptations can occur in concurrently training athletes in response to a 5-week training program, and these adaptations were possibly associated with the force and velocity characteristics of the training exercises. Angelik et al. [5] have studied the rate of force development (RFD) and muscle architecture early adaptations in response to training with fast- or slow-velocity eccentric squats. Eighteen young novice participants followed 6 weeks (two sessions/week) of either fast-velocity (Fast) or slow-velocity (Slow) squat eccentric-only training. The results of this study suggest that fast eccentric resistance training may be more appropriate for increases in rapid force production compared to slow eccentric resistance training, and this may be partly due to increases in muscle FL induced by fast eccentric training. Moreover, the main muscle architecture parameters, which were assessed through muscle ultrasound, had demonstrated some degree of alteration in the quadriceps femoris and gastrocnemius medialis, and each of these parameters may be theoretically useful for detecting the loss of muscle mass and functionality in geriatric patients [50].

However, so far, almost no studies compared the effects of football and running training interventions on the age-related decline of muscle strength and structure. This is the first study that aims at investigating the differential effects of football and running training response on muscle architecture, patella tendon properties, and thigh muscles. The proposed study should benefit participants’ physical, psychological, and social dimensions. It is assumed that running and football training not only may result in the improvement of the aerobic capacity and systemic arterial blood pressure reductions, but also can improve the functional parameters, including increased explosive power strength, balance, agility, and functional capacity. As our primary hypothesis, we expect moderate differences between both groups in muscle architecture CSA, FL, MT, PA, knee ROM, passive resistive torque, work, power, and tendon length and thickness as football entails are broader and multimodal muscular stimulus compared to aerobic training alone. These may lead to overall decrease in obesity risk and stress, and it could also prevent heart diseases, maintain muscle mass, and reduce the risk of depression. Moreover, the training programs may enhance muscle architecture and tendon thickness that causes a perceptible decrease in the probability of dynapenia and sarcopenia in the future.

## Trial status

The recruitment will start on 1 February 2020 and end on 15 March 2020.

Protocol version Number: 05.

## Abbreviations

VM: Vastus medialis
VL: Vastus lateralis
PA: Pennation angle
PT: Patella tendon
ROM: Range of motion
FL: Fascicle length
MT: Muscle thickness
CSA: Cross-section area
ML: Muscle length
HR: Heart rate
VO2 max: Maximal oxygen uptake
SPIRIT: Standard Protocol Items: Recommendations for Interventional Trials
